# Supplementary material for: The causal relationship between the human gut microbiota and pyogenic arthritis: a Mendelian randomization study
Source: Front Cell Infect Microbiol. 2024 Nov 26;14:1452480. doi: 10.3389/fcimb.2024.1452480 (PMC11629706; doi:10.3389/fcimb.2024.1452480)
Supplement: Supplementary file 1 [file DataSheet1.zip › mendelian test/ebi-a-GCST90027483.csv_leave_one_out.pdf]

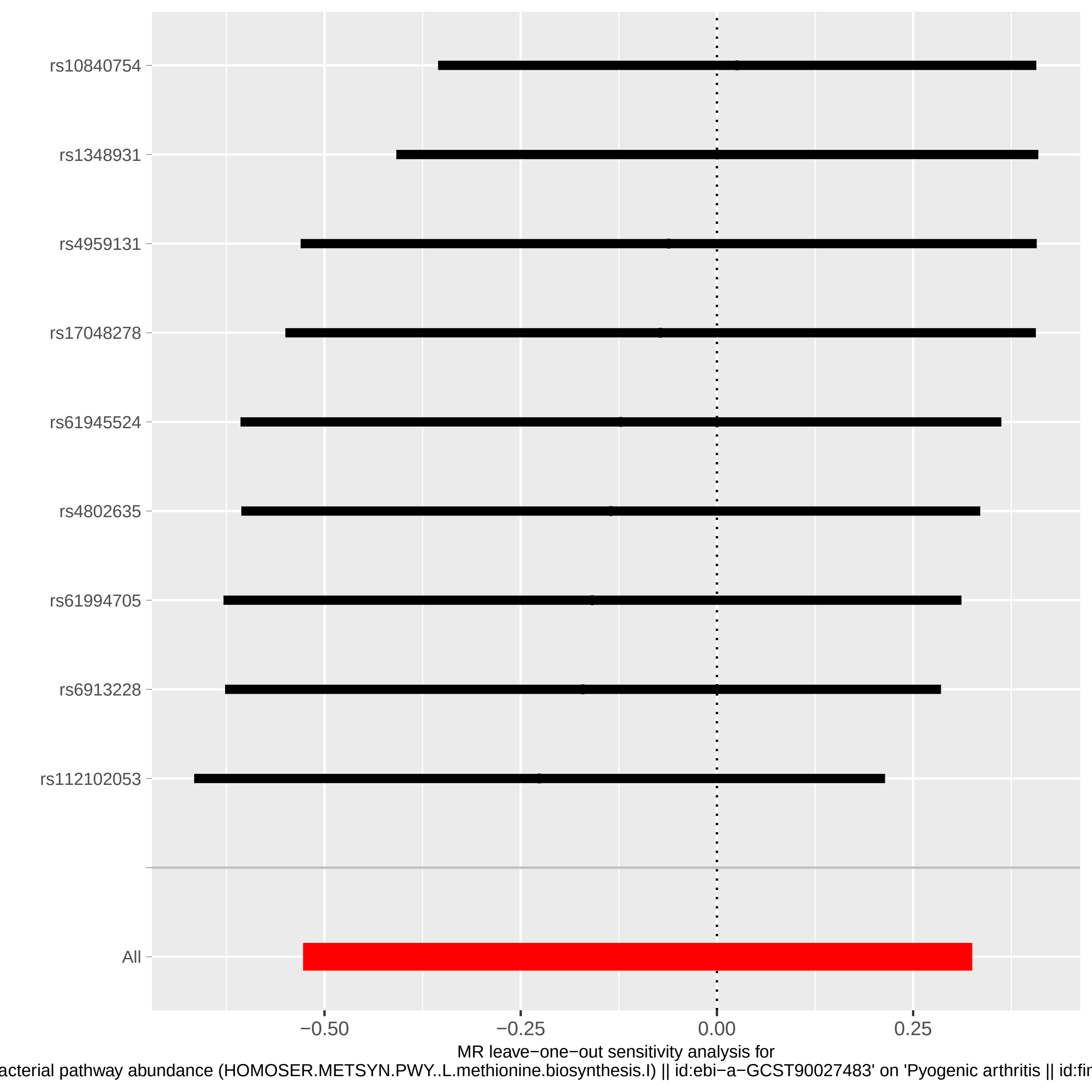
rs10840754
rs1348931
rs4959131
rs17048278
rs61945524
rs4802635
rs61994705
rs6913228
rs112102053
All
−0.50
−0.25
0.00
0.25
MR leave−one−out sensitivity analysis for
acterial pathway abundance (HOMOSER.METSYN.PWY..L.methionine.biosynthesis.I) || id:ebi−a−GCST90027483' on 'Pyogenic arthritis || id:fir
